# Correction: Musielak et al. CA-170 – A Potent Small-Molecule PD-L1 Inhibitor or Not? *Molecules* 2019, *24*, 2804

**DOI:** 10.3390/molecules30081816

**Published:** 2025-04-18

**Authors:** Bogdan Musielak, Justyna Kocik, Lukasz Skalniak, Katarzyna Magiera-Mularz, Dominik Sala, Miroslawa Czub, Malgorzata Stec, Maciej Siedlar, Tad A. Holak, Jacek Plewka

**Affiliations:** 1 Faculty of Chemistry, Jagiellonian University, Gronostajowa 2, 30-387 Krakow, Poland; 2 Department of Clinical Immunology, Institute of Pediatrics, Jagiellonian University Medical College, Wielicka 265, 30-663 Krakow, Poland

## Text Correction

There was an error in the original publication [1], where reference [36] was mistakenly referring to the wrong patent.

The following correction has been made to the first sentence of paragraph 5 in Section 1 “Introduction”:

“Aurigene and Curis have not disclosed the structures of **CA-170**, however, based on the analysis of Aurigene patents and the fact that it is derived from the **AUNP-12** peptide with known structure [35], **CA-170** is most likely compound 4 from the patent WO2015033299A1 [36], being a peptidomimetic, composed of an L-serine, D-asparagine, and l-threonine connected via diacylhydrazine and urea linker moieties, as presented in Figure 1 B.”

The following correction has also been made to the seventh sentence of paragraph 1 in Section 3 “Discussion”:

“Based on the patent and review analysis, we conclude that it is compound 4 from the patent WO2015033299A1 [36].”

## Reference Correction

The corrected reference [36] is as follows:

“36. Sasikumar, P.G.N.; Ramachandra, M.; Naremaddepalli, S.S.S. WO2015033299A1 2015. Available online: https://patents.google.com/patent/WO2015033299A1 (accessed on 5 June 2019).”

The authors state that the scientific conclusions are unaffected. These corrections were approved by the Academic Editor. The original publication has also been updated.
